# Highly Efficient and Stable White Light‐Emitting Diodes Using Perovskite Quantum Dot Paper

**DOI:** 10.1002/advs.201902230

**Published:** 2019-10-29

**Authors:** Chieh‐Yu Kang, Chun‐Ho Lin, Chih‐Hao Lin, Ting‐You Li, Sung‐Wen Huang Chen, Chun‐Lin Tsai, Chin‐Wei Sher, Ting‐Zhu Wu, Po‐Tsung Lee, Xuezhu Xu, Maolin Zhang, Chih‐Hsiang Ho, Jr‐Hau He, Hao‐Chung Kuo

**Affiliations:** ^1^ Department of Photonics and Institute of Electro‐Optical Engineering College of Electrical and Computer Engineering National Chiao Tung University Hsinchu 30010 Taiwan Republic of China; ^2^ Computer, Electrical, and Mathematical Sciences and Engineering Division King Abdullah University of Science and Technology (KAUST) Thuwal 23955‐6900 Saudi Arabia; ^3^ Raysolution LLC San Jose CA 95129 USA; ^4^ Department of Materials Science and Engineering City University of Hong Kong Kowloon Tong Hong Kong

**Keywords:** light‐emitting diodes, paper, perovskites, quantum dots, stability

## Abstract

Perovskite quantum dots (PQDs) are a competitive candidate for next‐generation display technologies as a result of their superior photoluminescence, narrow emission, high quantum yield, and color tunability. However, due to poor thermal resistance and instability under high energy radiation, most PQD‐based white light‐emitting diodes (LEDs) show only modest luminous efficiency of ≈50 lm W^−1^ and a short lifetime of <100 h. In this study, by incorporating cellulose nanocrystals, a new type of QD film is fabricated: CH_3_NH_3_PbBr_3_ PQD paper that features 91% optical absorption, intense green light emission (518 nm), and excellent stability attributed to the complexation effect between the nanocellulose and PQDs. The PQD paper is combined with red K_2_SiF_6_:Mn4^+^ phosphor and blue GaN LED chips to fabricate a high‐performance white LED demonstrating ultrahigh luminous efficiency (124 lm W^−1^), wide color gamut (123% of National Television System Committee), and long operation lifetime (240 h), which paves the way for advanced lighting technology.

## Graphical abstract

Perovskite quantum dot paper, a new type of perovskite quantum dot film, is demonstrated. Using a simple, fast, scalable, and inexpensive paper fabrication process, the resulting perovskite quantum dot paper is uniform, of high quality, and very stable; it is able to bear high energy radiation and greatly improve the efficiency of perovskite quantum dot–based white light‐emitting diodes.

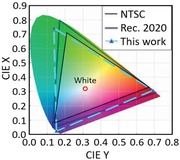

## 1 Introduction

Over the past decade, significant effort has been devoted to the development of highly efficient and long‐lasting white light‐emitting diodes (LEDs) for room lighting and display technologies.^1^, ^2^, ^3^ Emerging after conventional semiconductor and organic LEDs, colloidal quantum dot (QD) LEDs are considered the next step forward in the display market due to their narrow emission spectra, tunable emission wavelength, and small dimensions, which play a key role in shrinking the pixel sizes for micro‐LED displays.^4^, ^5^, ^6^, ^7^, ^8^ Among a variety of QDs, the hybrid halide perovskite (MAPbX_3_, MA = CH_3_NH_3_^+^; X = Cl^−^, Br^−^, or I^−^) QD is a promising candidate for next‐generation lighting applications due to its high quantum yield and impressive color purity, particularly at green wavelengths.^6^, ^9^, ^10^ The quantum yield of liquid phase perovskite QDs (PQDs) is typically greater than 80%, with the record high reaching 100% efficiency.^11^, ^12^, ^13^ Additionally, most PQDs feature a full width at half maximum (FWHM) of less than 30 nm,^7^, ^14^ which is favorable for improving the color purity of displays.^15^

To date, various PQD‐based white LEDs have been reported using diverse structural designs that feature different advantages and disadvantages. Due to the instability of PQDs under moisture, thermal, and high energy radiation conditions, it is crucial to utilize them in efficient and stable LED structures. Generally, PQDs serve as either the active light‐emitting material^16^, ^17^ or the color converter^18^, ^19^ in LEDs. However, previous studies have demonstrated that the operation lifetime of converter‐type PQD LEDs is significantly better than devices based on the active light emission mechanism because PQDs are fairly unstable under continuous electrical excitation.^17^ Therefore, we utilized a converter‐type structure for the white LED design in this study.

Converter‐type PQD LEDs can be further classified into three categories, including QD enhancement film (QDEF), QD color filters, and on‐chip QDs (Figure S1, Supporting Information). QDEF has already been mass produced by several TV manufacturers, in which large‐area PQD films are placed over the entire display.^20^, ^21^ However, QDEF LEDs are costly due to the large amounts of PQDs used. The QDEF structure can also lead to high optical loss >10%, which suppresses the device efficiency.^22^ Compared with QDEF, the PQD color filter design features higher efficiency; however, it is not easy to solve the cross‐talk issue between the red, green, and blue (RGB) color filters.^22^

On‐chip QD LEDs are preferable in display applications because of their high device performance, low production cost, and simple fabrication process, in which the QDs are coated on a blue LED chip or the top of the LED package.^9^, ^18^, ^19^, ^23^, ^24^, ^25^, ^26^ However, the high energy radiation of blue LEDs can lead to thermal quenching and photodegradation of the PQDs, thus hindering their practical application.^27^ These issues also cause the low luminous efficiency of on‐chip PQD LEDs (≈50 lm W^−1^), which is much lower than that of conventional phosphor‐based LEDs.^23^, ^27^ Controlling the uniformity and QD density of the QD film is also challenging.^28^ Spin‐coating is a conventional fabrication method; however, it is limited to wafers and leads to high production cost due to material waste during the coating process.^29^ Ink‐jet printing is also problematic due to slow production rates and the difficulty in fabricating pixel sizes of <10 µm.^15^ As a result, there is a need for a PQD film that can be made using cost‐effective and scalable manufacturing techniques and is also sufficiently stable to bear high energy radiation to improve the luminous efficiency of the on‐chip PQD LED.

To achieve these aims, we demonstrate a paper fabrication process using cellulose nanocrystals (CNCs) to produce a new type of PQD film, which we call PQD paper. CNC is naturally organized in an ordered crystalline structure, which provides strong mechanical strength to the paper. Moreover, the capping ligands of CNC play a key role in ligand‐assisted reprecipitation to confine the growth of the perovskite to QD structures.^30^ Using a simple, fast, scalable, and inexpensive paper fabrication process, the resulting PQD paper is uniform, of high quality, and stable, providing an excellent material for advanced PQD LEDs.

Specifically, we fabricated CH_3_NH_3_PbBr_3_ PQD paper possessing a peak emission wavelength at 518 nm and a narrow FWHM of 28 nm, which is suitable for acting as a green color converter. By incorporating the PQD paper with red K_2_SiF_6_:Mn^4+^ (KSF) phosphor and blue LEDs, we are able to achieve white LEDs featuring a wide color gamut of 123% of the National Television System Committee (NTSC) standard and an ultrahigh luminous efficiency of 124 lm W^−1^, which meets the efficiency requirement for commercial application (100 lm W^−1^). In the PQD paper, the capping ligands of the CNC (—HSO_3_^−^ and —O^−^) complex with the PQDs in the paper structure, which improves their stability. As a result, the PQD paper‐based LED is able to maintain 87.6% luminous flux after continuous operation for 240 h. Furthermore, benefiting from the flexible nature of paper, the use of curved PQD paper can further increase the viewing angle of the LED from 120° to 143° compared to the normal flat design. With superior optical properties, excellent stability, robust flexibility, and the capability to realize highly efficient emission, the PQD paper exhibits excellent potential for future solid‐state lighting.

## 2 Results and Discussion

**Figure 1:**
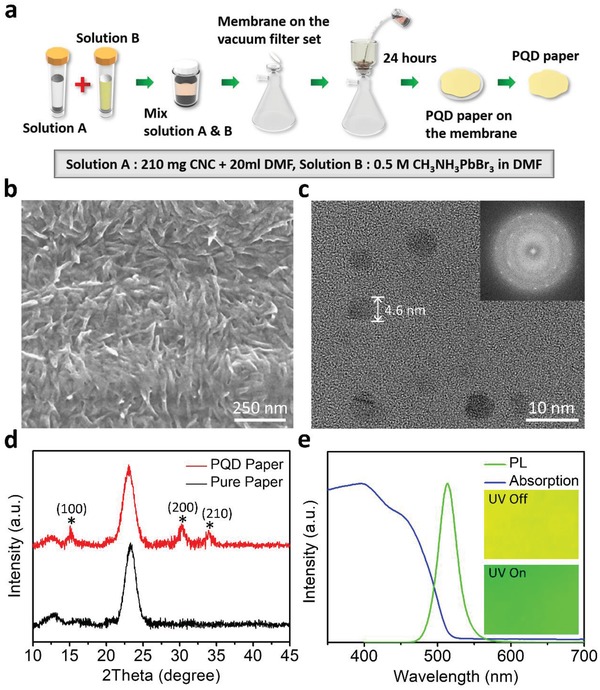
Fabrication and characteristics of the PQD paper. a) Schematic of the fabrication process of the PQD paper. b) SEM image of the PQD paper surface. c) TEM image of the CH_3_NH_3_PbBr_3_ PQDs obtained from the paper. The electron diffraction pattern in the inset reveals the high crystallinity of the PQDs. d) XRD patterns of the PQD paper and pure CNC paper. e) PL and UV–vis absorption spectra of the PQD paper. The insets show photographs of the PQD paper when the UV illumination is switched on and off.

The fabrication process of the PQD paper is shown in **Figure** **1**a. First, we mixed a CNC suspension and CH_3_NH_3_PbBr_3_ in dimethylformamide (see the Experimental Section for details). The CNC/perovskite mixture was then filtered and dried on the membrane for 24 h. During the drying process, the abundant —HSO3^−^ and —O^−^ capping ligands of the CNC confine the crystallization of the perovskite to QD structures through ligand‐assisted reprecipitation.^30^ The resulting PQD paper can be easily peeled off from the membrane.

Using scanning electron microscopy (SEM), we observed the surface morphology of the PQD paper, which demonstrated the entangled CNC structure (Figure 1b). These CNCs provide the PQD paper with unique mechanical strength and flexibility. Furthermore, the cross‐sectional SEM image in Figure S2 (Supporting Information) reveals that the thickness of the PQD paper is ≈45 µm. By adding more CNC and perovskite precursors with the same concentration, the thickness of PQD papers can be increased. However, preparing PQD paper with thickness less than 45 µm is challenging because thinner PQD paper becomes brittle and may break easily. Figure S3 (Supporting Information) shows the surface roughness of PQD paper measured by zygo profilometer. Because CNCs have much smaller dimension than normal celluloses, the root‐mean‐square surface roughness of PQD paper is 372 nm, which is much lower than that of conventional paper (surface roughness in microscale). By using a sequence of mechanical pressing methods during paper fabrication,^31^ it is possible to further improve the surface roughness of PQD paper for applications that require smooth surface. We observed the appearance of the PQDs using transmission electron microscopy (TEM), as shown in Figure 1c and Figure S4 (Supporting Information). The sizes of the PQDs were ≈3–8 nm, which should provide a strong quantum confinement effect and enhance the light emission of the perovskite. The X‐ray diffraction (XRD) patterns of the CH_3_NH_3_PbBr_3_ PQD paper and pure CNC paper are shown in Figure 1d. Both samples show strong diffraction peaks at 23°, which is caused by the CNC material; while the PQD paper reveals other peaks appearing at 15°, 30°, and 34°, which we assigned to the (001), (200), and (210) crystal planes of the CH_3_NH_3_PbBr QDs,^32^, ^33^ confirming the high purity of the PQDs in the paper.

Figure 1e shows the photoluminescence (PL) emission and ultraviolet–visible (UV–vis) absorption spectra of the CH_3_NH_3_PbBr_3_ PQD paper. The PQD paper exhibits bright green PL emission with an FWHM of 28 nm and a peak wavelength of 518 nm that corresponds to the sharp absorption edge cut‐off of the PQD paper. The strong absorption in the short wavelength region confirms the capability of the PQD paper to act as a color converter for blue GaN LED chips. The insets in Figure 1e are optical images of the PQD paper with and without UV excitation, demonstrating its good color uniformity in normal and PL conditions. Additionally, using a 450 W xenon lamp and spectrometer, we measured the optical absorption of the PQD paper to be 91% and the corresponding quantum yield to be 63.9% (Table S1, Supporting Information), which is not as high as pure PQDs because the CNCs absorb light in the UV region, thus suppressing the quantum yield.

Another substantial property of the PQD paper is its flexibility. We calculated its bending curvature using the equation^34^ $\text{Curvature}\;=\;\sqrt{24(s\;-\;d)}/d^{3/2}$, in which *s* is the initial length of the PQD paper and *d* is the horizontal distance between two edge points of the PQD paper at different bending conditions. Figure S5 (Supporting Information) demonstrates the PQD paper under different bending curvatures from 0.128 to 0.283 mm^−1^, which confirms its flexibility and compatibility for working on different curved surfaces.

**Figure 2:**
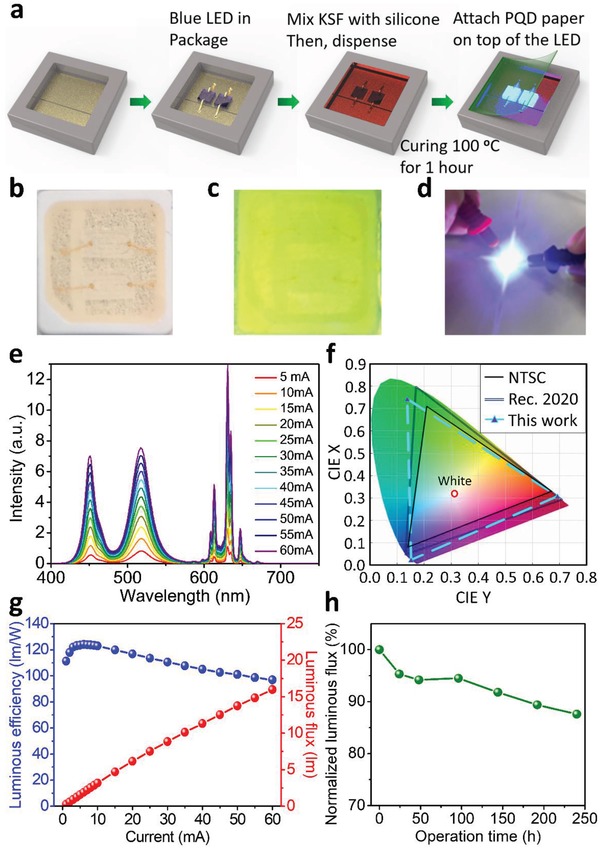
Fabrication and EL performance of the PQD paper‐based white LED. a) Schematic of the fabrication process of the PQD paper‐based LED. Photographs of b) the LED after KSF and silicone encapsulation, c) the completed PQD paper‐based LED, and d) the LED emission. e) EL spectra of the PQD paper‐based LED at different drive currents. f) CIE diagram illustrating the color gamut of the NTSC standard, the Rec. 2020 standard, and the PQD paper‐based LED. g) Current‐dependent luminous efficiency and luminous flux of the PQD paper‐based LED. h) Time‐dependent luminous flux of the LED device under continuous operation.

We used the PQD paper as the green color converter for a white LED. The fabrication process of the PQD paper based LED is shown in **Figure** **2**a. First, two 450 nm blue LED chips were packaged in a 3 mm × 3 mm LED unit. Next, we mixed KSF red phosphor with silicone resin and dispensed the mixture into the package (Figure 2b), as KSF can generate an emission spectrum consisting of several sharp peaks (FWHM < 5 nm) at ≈630 nm with 98.8% high color purity.^35^, ^36^ Moreover, previous studies have demonstrated that green PQDs with KSF phosphors possess a higher efficiency and wider color gamut than with other red QD phosphors.^20^, ^23^, ^24^, ^25^, ^27^ After 1 h curing, the PQD paper was attached on top of the package as the green color converter to achieve the white LED (Figure 2c,d), which features a proven on‐chip QD device design.^19^, ^27^, ^37^ However, due to the instability of the PQDs under high energy blue light illumination, most reported PQD white LEDs only show a luminous efficiency of ≈50 lm W^−1^,^23^, ^27^ which requires further improvement for practical use in room lighting (>100 lm W^−1^).^38^

The fabricated PQD paper based white LED can be immediately lit up by external current injection (Figure S6, Supporting Information). Figure 2e reveals the electroluminescence (EL) spectra of the PQD paper based device at different drive currents (from 5 to 60 mA), demonstrating the presence of three primary peaks located at 452, 518, and 630 nm, which correspond to the blue LED chips, the green PQD paper, and the red KSF, respectively (normalized EL spectra under different current are shown in Figure S7a of the Supporting Information). The FWHMs for the blue, green, and red emission peaks were 16, 28, and 5 nm, which enables strong white light emission with a correlated color temperature of 6706 K and color coordinate of (0.311, 0.320). The color rendering index (CRI) of PQD paper based white LED is 64. Conventional white light sources pursue emitter spectrum as broad as sunlight to achieve high CRI. On the other hand, the new‐generation QD‐based LED lighting system makes use of RGB cluster emitters with narrow bandwidth emission, which produces white color in different way against CRI system. Therefore, QD‐based LED can generate much purer RGB color and exhibit higher NTSC than conventional white light sources.

Figure 2f illustrates the color gamut of the PQD paper based LED, which covers a large color space of 123% of the NTSC standard and 92% of Rec. 2020, the most important color standard for next‐generation 8K4K displays.^18^ The color coordinates of the blue LED and KSF are very close to the blue and red points defined by Rec. 2020. However, more efforts are needed to move the color coordinate of the green PQD paper (0.138, 0.743) to the ideal green point of the Rec. 2020 standard (0.170, 0.797). The current‐dependent luminous efficiency and flux of the PQD paper based LED are shown in Figure 2g. The maximum efficiency of the PQD paper based LED is 124 lm W^−1^ at 6 mA, which is much higher than typical PQD LEDs. Even when the drive current goes up to 50 mA, the PQD paper based LED still exhibits a luminous efficiency of over 100 lm W^−1^, indicating the device can maintain its high performance at different drive currents. Furthermore, the PQD paper based LED displays excellent stability. After continuous operation of 240 h, the device shows just 12.4% degradation of the luminous flux (Figure 2h and Figure S7b, Supporting Information).

**Figure 3:**
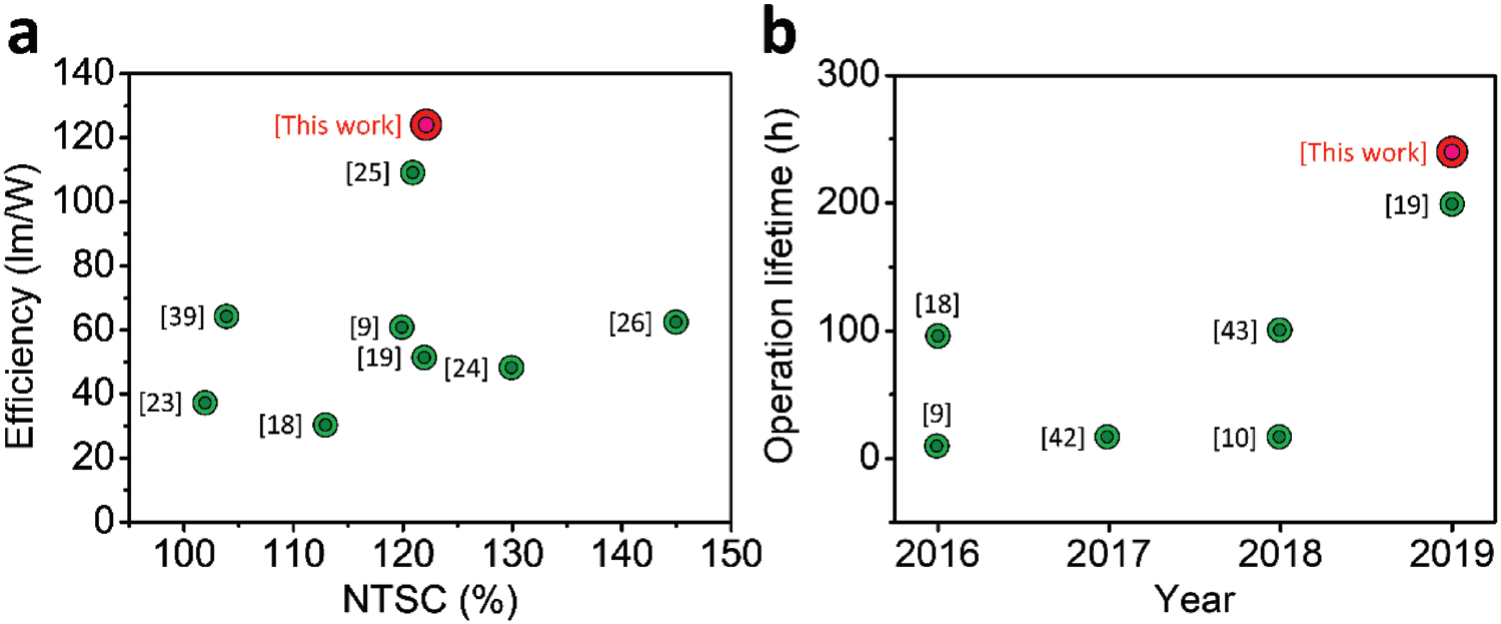
Comparison of the device performance between our PQD paper‐based LED and other reported LEDs using QDs as the color converter. A summary of a) the luminous efficiency and color gamut performance and b) operational durability of QD‐based LEDs reported in previous studies and this work.

**Figure** **3**a summarizes the luminous efficiency and color gamut performance of converter‐type QD LEDs reported in the literature and this work (the details of these previous studies are listed in Table S2 of the Supporting Information).^9^, ^18^, ^19^, ^23^, ^24^, ^25^, ^26^, ^39^ Among nonperovskite QD LEDs, liquid phase CdSe QDs have shown the highest performance with a luminous efficiency of 64 lm W^−1^, reported by Sadeghi et al.^39^ However, because green CdSe QDs feature a longer PL wavelength (≈550 nm) than the ideal green wavelength (≈525 nm), LEDs based on CdSe QDs are not conducive to achieving a wide color gamut. Meanwhile, due to the poor thermal stability of PQDs,^12^ most PQD‐based LEDs exhibit efficiencies of below 70 lm W^−1^. Embedding PQDs into hard porous templates or polymeric matrices has been proposed as an effective way to improve the stability.^25^, ^40^, ^41^ As an example, Zhou et al. reported a polyvinylidene fluoride/PQD composite film based white LED that can achieve a high efficiency of 109 lm W^−1^ and NTSC of 121%.^25^ In our work, the PQD paper enables an LED luminous efficiency that is further improved to 124 lm W^−1^ and with a color gamut that reaches 123% of the NTSC standard. Most importantly, the studied LED exhibits a long operation lifetime of 240 h, which is much longer than other PQD‐based white LEDs (Figure 3b).^9^, ^10^, ^18^, ^19^, ^42^, ^43^

The enhanced performance of our PQD paper LED device can be attributed to the QD structure within the cellulose paper. Surface engineering using controlling ligands to generate strong complexation on PQD surface is proven a useful technique for improving PQD stability.^12^ For typical colloidal PQD synthesis, long chain oleic acid (OA) and oleylamine (OLA) are the most commonly used capping ligands at PQD surface. However, OA and OLA ligands are not tightly bound to the PQD surface and can be easily lost during the purification process or colloidal to solid transformation, which is regarded as a key reason for the PQD instability.^44^ As a result, developing a compact ligand layer has become a hot research topic for PQD to minimize the ligand loss. For example, Li et al. employed stearic acid and octadecylamine to cap the PQDs. The resulting PQDs retain 80% of initial PL intensity after storing in the air for 30 d, while OA and OLA capped counterpart shows 40% intensity in the same condition.^45^ Similarly, long chain didodecyl dimethylammonium sulfide,^46^ zwitterionic,^47^ and bidentate^48^ ligands were utilized to produce strong complexation on PQD surface, and the stability of PQDs was significantly increased. Based on this prospective, crosslinking surface ligands such as 4‐vinylbenzyldimethyloctadecylammonium chloride and trimethylaluminum were also introduced to strengthen the surface interactions.^49^, ^50^ Owing to the crosslinking structures, these materials can greatly reduce the ligand loss, and the resulting PQDs successfully maintain high performance for a few months.

In this study, CNCs with strong mechanical strength and entangled cellulose structure can act as natural crosslinking ligands and make the entire PQD paper robust and stable.^51^ CNCs contain very electronegative —HSO_3_^−^ and —O^−^ capping ligands, which tend to complex with cations in the PQDs (CH_3_NH_3_^+^, Pb_2_^+^), thus enhancing the PQD stability.^52^ It is worth noting that the synthesis method of PQD paper is unique from convention colloidal PQD synthesis. For colloidal PQD, OA and OLA capping ligands are added into the precursor solution to confine the growth of perovskite in QD size. For PQD papers, the growth of PQDs is under continuous vacuum suction with an assist of surrounding CNC capping ligands to confine the perovskite growth, and the resulting PQDs are in solid forms. Therefore, the capping ligand loss during PQD purification and colloidal to solid transformation can be totally avoided, leading to well‐capped and stable PQDs. The X‐ray photoelectron spectroscopy (XPS) spectra of PQD paper are shown in Figure S8 (Supporting Information). Because the volume of CNCs is much larger than PQDs in the paper structure, the O‐1s and C‐1s peaks from CNCs dominate the XPS spectrum. However, the high‐resolution XPS scans reveal the Br‐3d, Pb‐4f, and N‐1s peaks, confirming the presence of PQDs. Generally, the Pb‐4f peak of PQDs using ligand‐assisted synthesis tends to shift to lower binding energy compared with normal bulk perovskite single crystals due to the interfacial electron transfer from electron‐rich surface capping ligand to Pb^2+^ cation.^53^, ^54^, ^55^ Herein, the CH_3_NH_3_PbBr_3_ PQDs based on cellulose‐assisted growth demonstrate Pb‐4f_7/2_ and Pb‐4f_5/2_ peaks at 138.3 and 143.2 eV (Figure S8c, Supporting Information), which is lower than that of normal CH_3_NH_3_PbBr_3_ perovskites (around 138.8 and 143.7 eV),^55^, ^56^, ^57^, ^58^ implying that the complexation effect occurs between CNCs and PQDs.

In addition, the gaps between individual CNCs can increase the heat dissipation area, which also improves the thermal stability of the PQDs, leading to even better thermal resistance than state‐of‐the‐art CdSe QDs (Figure S9, Supporting Information). While it may not be a good idea to operate the paper device at high temperature due to cellulose distortion, the good thermal resistance of the PQD paper is enough to dissipate the heat generated by high flux emission, allowing the device to be stably operated at room temperature. We compared the operation lifetime of the PQD paper based LED with other PQD LEDs in the literature, as shown in Figure 3b and Table S3 (Supporting Information).^9^, ^10^, ^18^, ^19^, ^42^, ^43^ Our PQD paper device reveals a much longer operation lifetime (240 h) than others, thus confirming the high stability of the PQD paper based LED and its potential for next‐generation PQD lighting applications.

**Figure 4:**
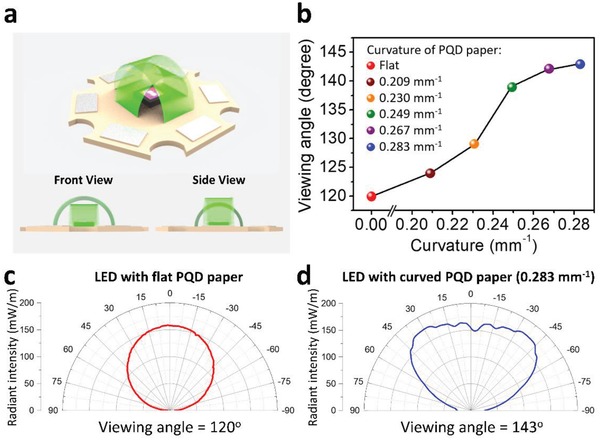
LED with a curved PQD paper color converter design. a) The schematic diagram of the LED featuring the curved PQD paper. b) The viewing angle of the LED as a function of the curvature of the PQD paper. Angular distribution of radiant intensity for c) an LED with a flat PQD paper converter, and d) an LED with a curved PQD paper (0.283 mm^−1^ curvature).

To improve the viewing angle of the PQD paper based LED, we designed a curved color converter structure for the LED device, as shown in **Figure** **4**a. The viewing angle is defined by the angle range where the LED brightness is above half of the maximum brightness. We found that the viewing angle of the LED increased from 120° to 143° as the curvature of the PQD paper increased from 0 mm^−1^ (flat status) to 0.283 mm^−1^ (Figure 4b). Figure 4c,d plots the angular distribution curves of radiant intensity for LEDs with a flat and curved PQD paper structure (0.283 mm^−1^ curvature). Compared to the flat device, the radiant intensity of the LED with the curved PQD paper is more uniform at different angles, and the viewing angle is larger, which is beneficial. For example, in the design of a direct backlight system, using LEDs with larger viewing angles can increase the pitch between LEDs and decrease the optical distance, which means we can reduce the usage of LEDs and fabricate thinner backlight displays for future mini‐LED backlight applications.

## 3 Conclusion

In summary, we have successfully developed PQD paper demonstrating impressive capability to realize white LEDs with high efficiency and wide color gamut, suggesting a promising material for display technology. The paper shows a high optical absorption of 91%, as well as uniform emission with an FWHM of 28 nm and a peak wavelength of 518 nm. The proposed white LED, composed of green PQD paper, red KSF phosphor, and blue LED chips, reveals a remarkable luminous efficiency of 124 lm W^−1^, a wide color gamut of 123% of the NTSC standard, and a viewing angle of 120°. The device also demonstrates superior stability, operating for 240 h with just 12.4% luminous degradation. In addition, the viewing angle of the LED can be further improved to 143° by using the flexible PQD paper as a curved color converter, thus illustrating the multifunctionality of the PQD paper.

The device demonstrated here follows the remote‐type phosphor LED design, which has been proven as more efficient and ideal LED structure than prototype design for PQD materials.^12^, ^19^, ^27^, ^37^ In remote‐type design, the installation of PQD layer on top of the LED package can avoid the direct contact between PQDs and high‐density heat inside the LED package, thus improving the stability and efficiency. According to current industry standard, the luminous efficiency of commercial device using red KSF and green nitride phosphor is around 110–120 lm W^−1^, and the NTSC is around 90%.^59^ The lighting performance of PQD paper based LED can meet the industry standard and the device stability is much better than other PQD‐based LEDs in the literature. However, from industry's point of view, the stability is still not good enough and should be further improved for commercial applications. Additionally, there are some other areas that can be addressed in the future. The color coordinates of the green PQD paper are still far from the ideal Rec. 2020 standard, and the PQD paper is too thick compared to conventional QD films, which may lead to lower transparency. However, research on the PQD paper is still in the infancy stage, and without any additives or additional engineering, the PQD paper has already shown the capability to realize efficient and stable LEDs, which is promising for future lighting applications.

## 4 Experimental Section

*Fabrication of the PQD Paper*: To prepare the cellulose suspension, 210 mg of freeze‐dried CNCs (Cellulose Lab, Canada) was mixed with 20 mL of anhydrous dimethylformamide solution (99.8%, Sigma), followed by sonication treatment for 2 h. For the perovskite solution, an equimolar amount of CH_3_NH_3_Br (98% Sigma) and PbBr_2_ (99%, Sigma) powders was dissolved in anhydrous dimethylformamide with a molar concentration of 0.5 m each and was stirred at 90 °C for 24 h. Then, 1 mL of perovskite solution was added to 7 mL of the CNC suspension and the solution was placed in a sonication bath for 2 h. The mixed solution was filtered through a filter membrane (20 nm pore size, Whatman) that was installed on a vacuum filter setup. The CNC/perovskite material was collected on the filter membrane and dried for 24 h under continuous vacuum pumping to obtain the PQD paper. Finally, the PQD paper was removed from the membrane.

*PQD Paper Characterization*: A cold field emission gun SEM (Hitachi Regulus 8220) with a beam resolution of 0.9 nm was employed to survey the surface morphology and thickness of the PQD paper. The surface roughness of PQD paper was measured using Zygo profilometer (NewView 7300). The appearance of the PQDs in the paper was examined using a TEM (Titan‐CT) operated at 300 kV. The TEM images were recorded by an ultrascan CCD camera from Gatan. The XRD patterns of the PQD paper and pure CNC paper were measured by a Bruker D8 Advance diffractometer. PL spectrum of the PQD paper was obtained using an Edinburgh Instruments Spectrofluorometer FS5 with a 150 W xenon lamp combined with an excitation monochromator. UV–vis absorption spectrum of the PQD paper was investigated using a spectrophotometer (Lambda 1050). XPS measurement was carried out using a Thermo Scientific K‐Alpha XPS system. For quantum yield and absorption measurements, a PL spectrometer (Spexflurolog‐3, Jobin Yvon Instrument) equipped with a 450 W xenon lamp and a photodetector (R928, Hamamatsu Photonics) were utilized.

*Fabrication of the PQD Paper Based LED*: Two 450 nm blue GaN LED chips (Epistar Corporation, Taiwan; chip size: 500 µm × 1000 µm) were mounted in a 3 mm × 3 mm package that consisted of a Ag‐plated lead‐frame and composite light reflector, followed by Au wire bonding to build the connection between the LED chips and electrodes. Next, KSF red phosphor (General Electric company, US) was mixed with silicone resin and the mixture was dispensed into the LED package. After 1 h curing, the PQD paper was then attached to the top of the package using silicone glue to complete the PQD paper based LED device.

*Characterization of the PQD Paper Based LED*: The EL measurements of the PQD paper based LED were carried out using a spectrometer CAS 140CT (Instrument System GmbH, Germany) equipped with a 50 cm integrating sphere. For viewing angle measurements, a goniophotometer LEDGON 100 (Instrument System GmbH, Germany) was employed to analyze the angle‐dependent radiation with 1° angular resolution.

## Conflict of Interest

The authors declare no conflict of interest.

## Supporting information

Supplementary
