# Supplementary material for: Highly Efficient and Stable White Light‐Emitting Diodes Using Perovskite Quantum Dot Paper
Source: Adv Sci (Weinh). 2019 Oct 29;6(24):1902230. doi: 10.1002/advs.201902230 (PMC6918104; doi:10.1002/advs.201902230)
Supplement: Supplementary file 1 — Supplementary [file ADVS-6-1902230-s001.pdf]

Copyright WILEY-VCH Verlag GmbH & Co. KGaA, 69469 Weinheim, Germany, 2019.

ADVANCED SCIENCE
Open Access

Supporting Information

for *Adv. Sci.,* DOI: 10.1002/advs.201902230

Highly Efficient and Stable White Light-Emitting Diodes Using Perovskite Quantum Dot Paper

Chieh-Yu Kang, Chun-Ho Lin, Chih-Hao Lin, Ting-You Li, Sung-Wen Huang Chen, Chun-Lin Tsai, Chin-Wei Sher, Ting-Zhu Wu, Po-Tsung Lee, Xuezhu Xu, Maolin Zhang, Chih-Hsiang Ho, Jr-Hau He,* and Hao-Chung Kuo**

Copyright WILEY-VCH Verlag GmbH & Co. KGaA, 69469 Weinheim, Germany, 2018.

Supporting Information

Highly Efficient and Stable White Light-Emitting Diodes Using Perovskite Quantum Dot Paper

Chieh-Yu Kang, Chun-Ho Lin, Chih-Hao Lin, Ting-You Li, Sung-Wen Huang Chen, Chun-Lin Tsai, Chin-Wei Sher, Ting-Zhu Wu, Po-Tsung Lee, Xuezhu Xu, Maolin Zhang, Chih-Hsiang Ho, Jr-Hau He* and Hao-Chung Kuo**

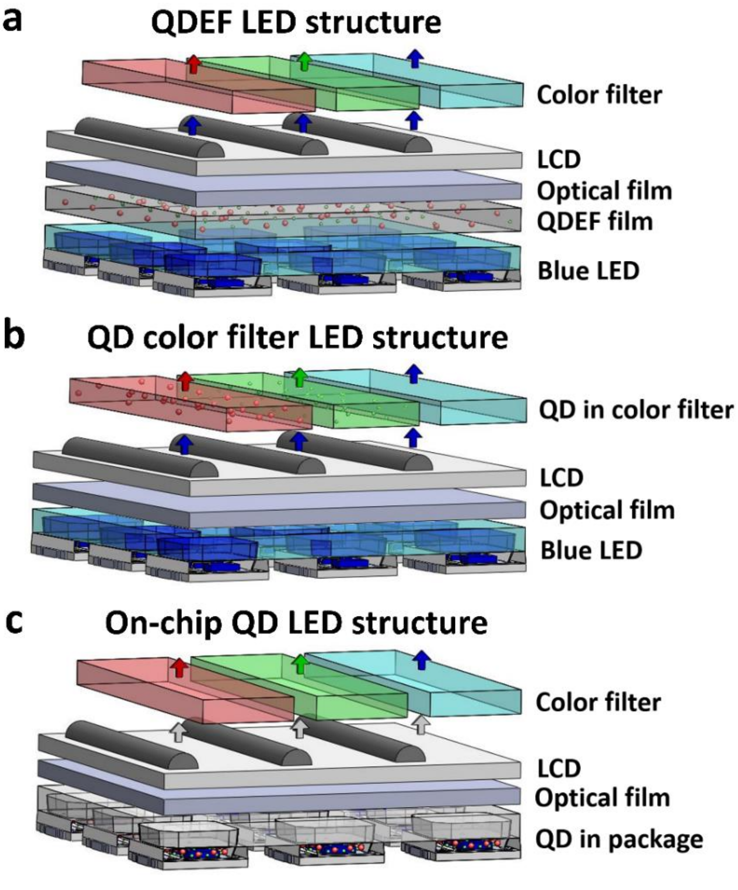

Figure S1. Structural schematic of different converter-type QD LEDs, including a) QDEF, b) QD color filter, and c) on-chip QD LED structures.

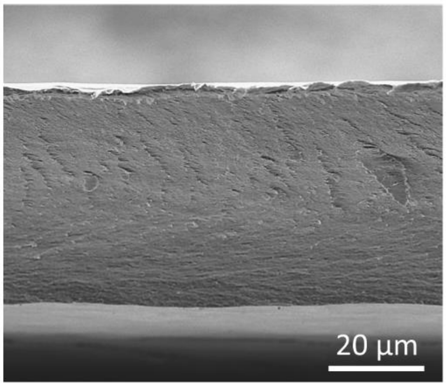

Figure S2. Cross-sectional SEM image, showing the thickness of the PQD paper is 45 μm.

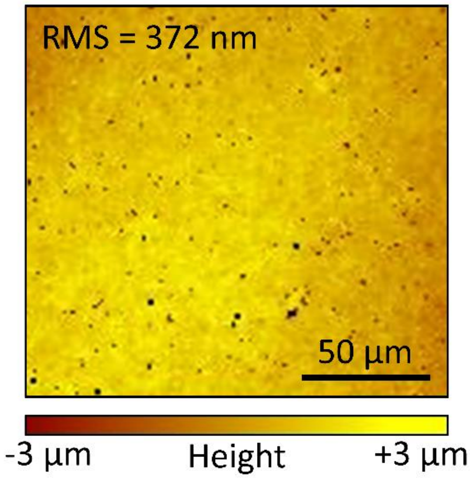

Figure S3. Surface morphology of PQD paper. The root-mean-square surface roughness (RMS) of PQD paper is 372 nm.

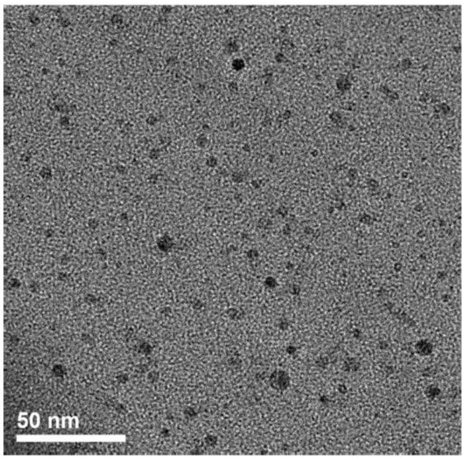

Figure S4. TEM image of the $CH_3NH_3PbBr_3$ QDs obtained from the PQD paper.

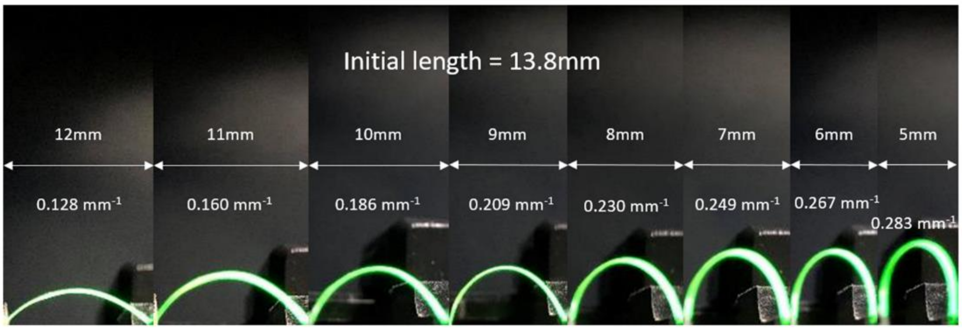

Figure S5. Optical photos of PQD papers under different bending curvatures.

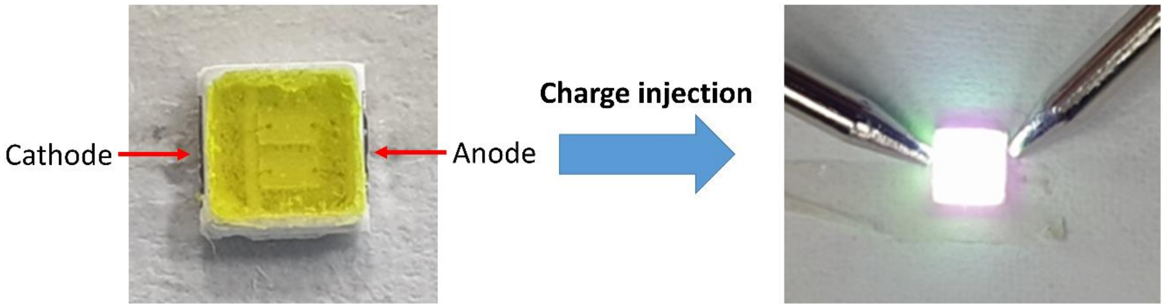

Figure S6. The PQD paper-based white LED can be immediately lit up by charge injection.

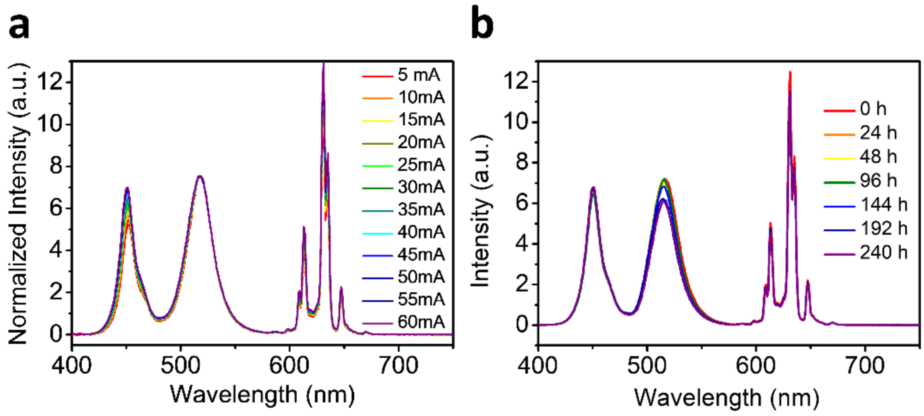

Figure S7. a) Normalized EL spectra of the PQD paper-based LED at different drive currents. b) EL spectra of the PQD paper-based LED under different operation durations.

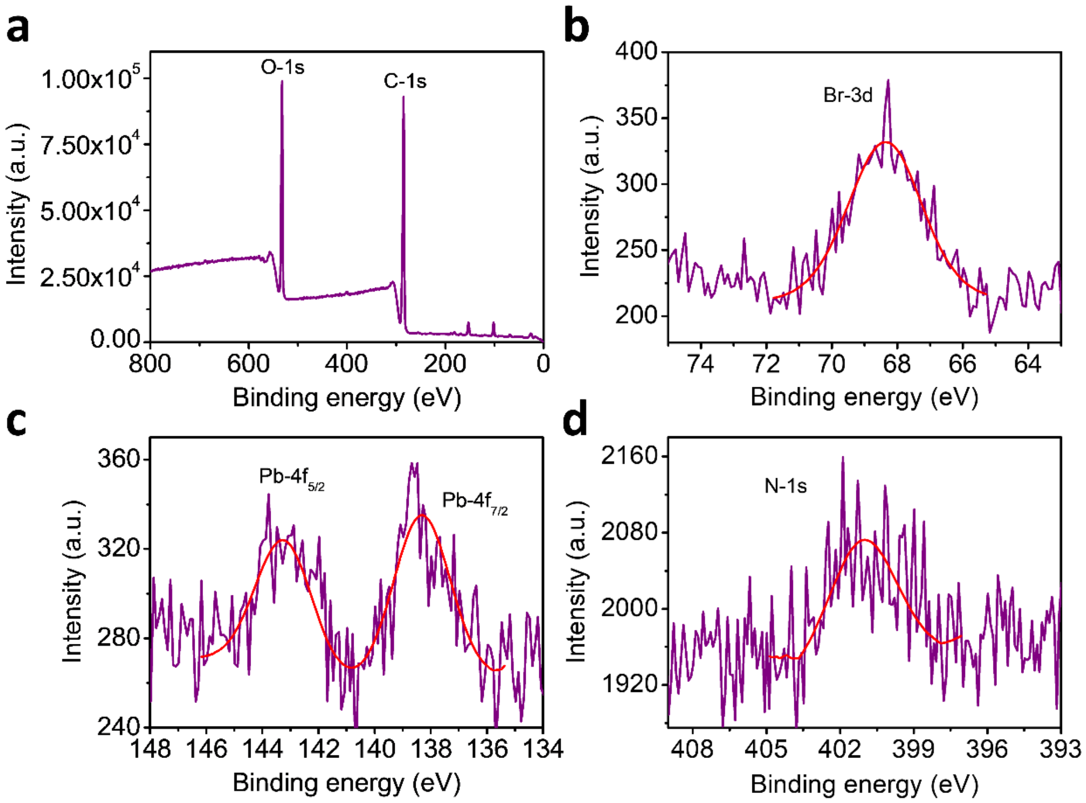

Figure S8. XPS spectra of PQD paper. a) Full XPS spectrum. b-c) High resolution Br-3d, Pb-4f, and N-1s XPS spectra.

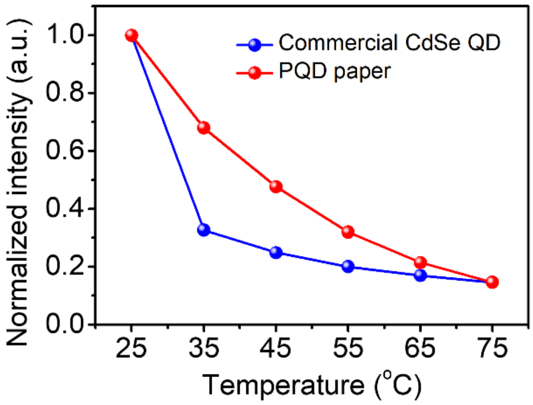

Figure S9. The normalized radiant intensity of the PQD paper-based and CdSe QD-based LEDs as a function of temperature, demonstrating the better thermal resistance of the PQD paper.

Table S1. Quantum yield of the PQD paper obtained by the PL spectrometer.

Absorption	91.1%
Transmission	8.9%
Absorbance	1.05
Quantum Yield	63.9%
Color	x = 0.1379, y = 0.7429

Table S2. The luminous efficiency and color gamut of QD-based white LEDs.

Ref	Year	Material	Quantum dot type in package	Efficiency (lm/W)	NTSC (%)
[18]	2016	519 nm green mesoporous PQD,<br>625 nm red PQD	Mixed in silicone resin	30	113
[23]	2016	523 nm mesoporous $CsPbBr_3$/SDDA in PMMA,<br>630 nm KSF	Powders type	37	102
[9]	2016	522 nm & 624 nm perovskite, QD/silica composites	Powders type	61	120
[24]	2015	515 nm colloidal $CH_3NH_3PbBr_3$ QDs, 630 nm KSF	PQD in PMMA, KSF in silicone,	48	130
[25]	2016	525 nm $MAPbX_3$/PVDF composite films, 630 nm KSF	PQD in PVDF, KSF in silicone,	109	121
[26]	2016	495 nm $CsPb(Br_{0.75}Cl_{0.25})_3$,<br>515 nm $CsPbBr_3$,<br>540 nm $CsPb(Br_{0.65}I_{0.35})_3$,<br>578 nm $CsPb(Br_{0.5}I_{0.5})_3$,<br>635 nm $CsPb(Br_{0.35}I_{0.65})_3$	Film type	62	145
[39]	2018	625 nm red CdSe/CdS/ZnS QD,<br>555 nm green CdSe/ZnS/CdSZnS QD	Liquid type	64	104
[19]	2019	519 nm Green PQD,<br>625 nm Red PQD	Liquid + solid type (hybrid)	51	122

Table S3. The operation lifetime and radiation performance of QD-based white LEDs.

Ref	Year	Material	Quantum dot type in package	Operation time (h)	Radiation remained
[18]	2016	519 nm green mesoporous PQD, 625 nm red PQD	Mixed in silicone resin	96	80%
[10]	2018	407 nm $Cs_3Bi_2Br_9$ QDs/silica composites, 551 nm $Y_3Al_5O_{12}$ phosphor	Powders type	16	78%
[9]	2016	522 nm & 624 nm perovskite QD/silica composites	Powders type	10	90%
[42]	2017	525 nm TDPA coated $CsPbBr_3$ QD, 630 nm KSF	Mixed in silicone resin	16	93%
[43]	2018	630 nm $CsPbBr_{1.2}I_{1.8}$ QD, 540 nm $Y_3Al_5O_{12}:Ce^{3+}$ phosphor	Liquid type	100	95%
[19]	2019	519 nm Green PQD, 625 nm Red PQD	Liquid + solid type (hybrid)	200	88%
